# Chronic Ulcer on the Tongue as a Manifestation of Histoplasmosis During Anti-tumor Necrosis Factor-Alpha (TNF-⍺) Inhibitor Therapy for Psoriasis

**DOI:** 10.7759/cureus.45217

**Published:** 2023-09-14

**Authors:** Iris Harrison, Mahtab Forouzandeh, Kiran Motaparthi

**Affiliations:** 1 Department of Dermatology, University of Florida College of Medicine, Gainesville, USA

**Keywords:** tumor necrosis factor-alpha, adalimumab, granuloma, ulcer, tongue, histoplasmosis

## Abstract

Anti-tumor necrosis factor-alpha (TNF-⍺) inhibitors are commonly used in the treatment of inflammatory conditions such as psoriasis. However, these agents lead to increased susceptibility to infections. We report a patient with reactivation of latent histoplasmosis six months after starting an anti-TNF-⍺ inhibitor for the treatment of psoriasis. Dermatologists should be aware of the risks associated with initiating therapy and maintain a low threshold of suspicion for this infection in patients on anti-TNF-⍺ inhibitors presenting with oral ulcers.

## Introduction

Tumor necrosis factor-alpha (TNF-⍺) inhibitors are commonly employed in the treatment of psoriasis [1]. These agents inhibit TNF-⍺ which is responsible for the development and maintenance of granulomas [2]. TNF-⍺ inhibitors suppress T-cell proliferation and alter the Th1 immune response [2]. Patients taking TNF-⍺ inhibitors are at increased susceptibility to infections, including histoplasmosis, a systemic infection caused by *Histoplasma capsulatum* [3].

## Case presentation

**Figure 1:**
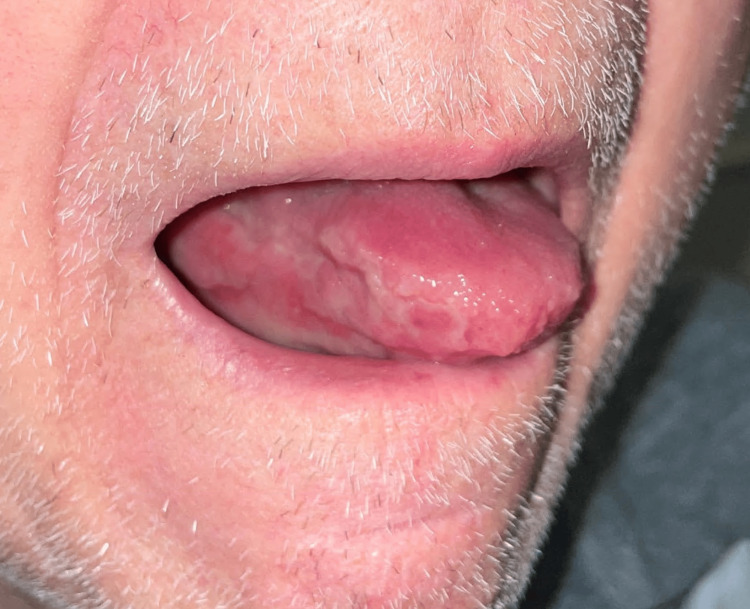
Chronic ulceration at the right side of the tongue.

**Figure 2:**
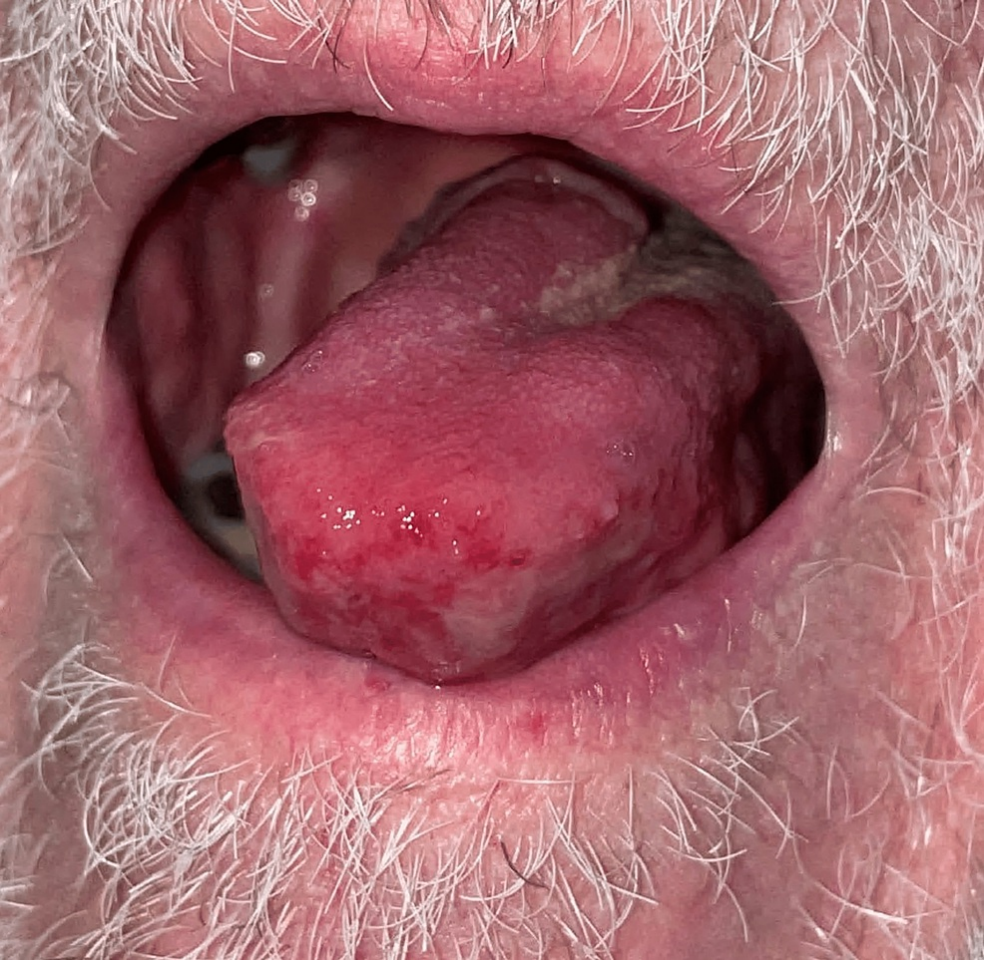
Chronic ulceration at the base of the tongue.

A 72-year-old man presented to the emergency department with an 18-month history of exquisitely painful ulcers at the base and right side of his tongue (Figures 1, 2) associated with odynophagia and dysphagia. He was unable to tolerate oral intake due to pain and reported a 50-pound weight loss over the course of four weeks. Two years prior to presentation, he had started treatment with subcutaneous adalimumab 40 mg every two weeks for psoriasis but had discontinued the adalimumab four months prior to presentation given concern the medication was contributing to his oral symptoms. He denied recent travel but previously lived in rural Panama, Honduras, and Nicaragua thirty years prior. Examination revealed tongue ulcers at the base and right side of his tongue without involvement of buccal mucosa or gingiva. Skin examination was clear without evidence of psoriasis.

**Figure 3:**
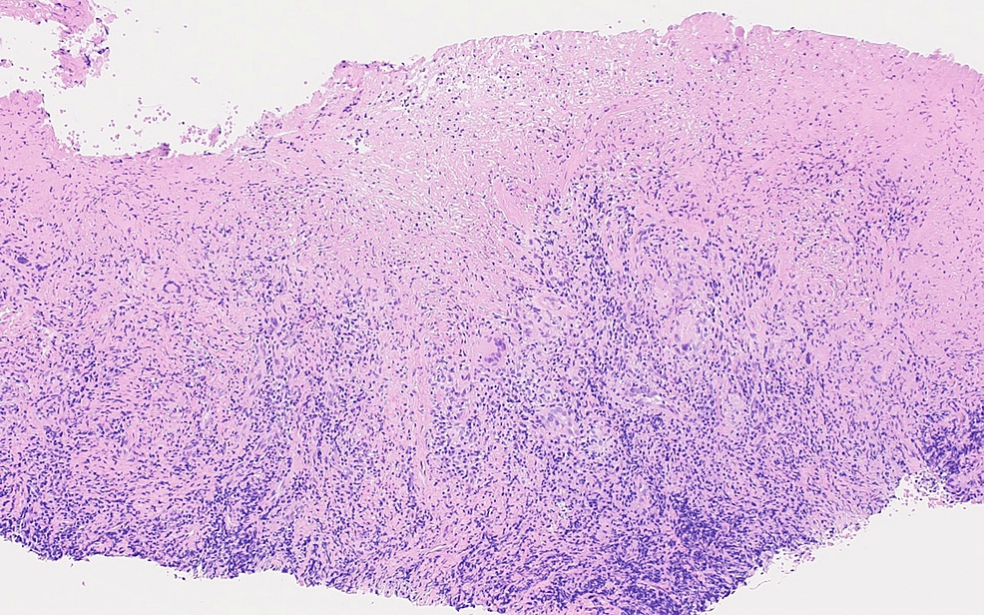
Necrotizing granulomas with ulceration and lymphocytic infiltrate (H&E, 100x magnification)

**Figure 4:**
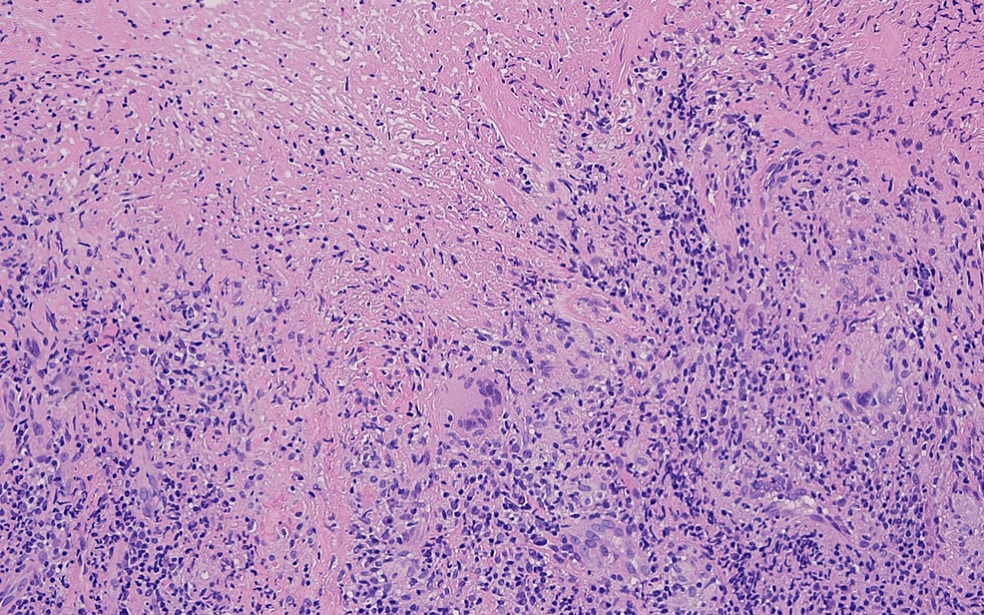
Necrotizing granuloma with multinucleate giant cells and lymphocytic infiltrate (H&E, 200x magnification)

He was hospitalized for inability to tolerate oral intake, pain control, and further workup for his tongue ulceration and weight loss. The tongue ulcer was biopsied for histopathologic evaluation. Hematoxylin-and-eosin (H&E) stain demonstrated necrotizing granulomas with ulceration and lymphocytic infiltrate (Figure 3) and multinucleate giant cells (Figure 4). Grocott’s methenamine silver stain demonstrated no evidence of fungal elements. No additional stains were performed. Indirect immunofluorescence was negative for antineutrophil cytoplasmic antibodies (ANCA). The ACE level was negative. Sedimentation rate, CRP, vitamin D, vitamin B12, folate, and ferritin were within normal limits. PCR was negative for HSV, VZV, CMV, and coxsackievirus A and B. Serologies for *T. pallidum*, hepatitis A, hepatitis B, hepatitis C, and HIV were negative. Interferon gamma release assay was negative. Chest X-ray (CXR) demonstrated a left lower lobe calcified nodule with multiple bilateral calcified granulomas. Due to concern of potential tonsillar abscess given odynophagia, computed tomography (CT) of the head and neck was performed but was unrevealing. Given substantial weight loss and concern for malignancy, CT of the chest and abdomen was completed but was also unremarkable. Upper endoscopy and colonoscopy to identify potential evidence of Crohn's disease were unrevealing. Serum cryptococcal antigen and urine *Histoplasma* antigen were negative. Acid-fast blood cultures, bacterial and acid-fast bacterial tissue cultures, and fungal tissue cultures demonstrated no growth to date.

During his admission and workup, he received treatment with a five-day course of intravenous dexamethasone 8 mg three times daily, viscous lidocaine for pain control, and oral nystatin. He demonstrated rapid clinical improvement with decreased pain in his tongue and was able to tolerate an oral diet. He received a steroid taper and was discharged six days later given his clinical improvement and negative workup at the time.

Three days later, fungal tissue culture demonstrated *H. capsulatum* after 10 days of growth on inhibitory mold agar. Acid-fast blood cultures and bacterial and acid-fast bacterial tissue cultures were negative. The patient was notified to return to the hospital for further workup and appropriate antifungal treatment. Upon admission, he received intravenous liposomal amphotericin B 3 mg/kg/day for two weeks. Further workup for histoplasmosis revealed positive serum *Histoplasma* antibodies. DNA sequencing confirmed that the *Histoplasma* isolate in this patient was a Latin American group A strain, also known as *H. suramericanum*. No tissue smear was performed for staining of *Histoplasma*. He was transitioned to oral itraconazole, with a loading dose of 200 mg three times daily for three days and then maintenance therapy of 200 mg daily. Given his clinical stability and resolution of symptoms, he was discharged with oral itraconazole.

At follow-up two months later, the patient reported symptomatic improvement in the ability to swallow soft foods and markedly reduced edema and pain; symptomatic improvement was correlated with a decrease in the size of the ulcer. His skin examination remained clear, so TNF-α inhibitor therapy was not reinstituted. He was continued on topical steroid treatment as needed for his psoriasis. He was scheduled for close follow-up with plans to continue oral itraconazole for 12 months or possibly convert to life-long itraconazole given his relatively immunocompromised state.

## Discussion

Histoplasmosis is a common mycosis caused by *H. capsulatum*. While classically endemic to the Ohio and Mississippi River Valleys of the United States, histoplasmosis is also endemic to Latin America [4]. It is associated with exposure to soil contaminated with bird or bat guano. Characteristically affected patients include cave explorers and farmers with chicken coops. Diagnosis relies on clinical presentation and epidemiology with laboratory culture, histopathology, and serology [5].

Disseminated histoplasmosis commonly occurs in immunosuppressed patients, including those patients undergoing treatment with TNF-α inhibitors [5]. TNF-α plays a role in the host defense against *Histoplasma capsulatum* [2]. Reactivation of latent histoplasmosis and subsequent dissemination following treatment with TNF-α inhibitors including adalimumab have been described [6,7]. In our patient, the calcified pulmonary granulomas on CXR indicate prior exposure to *Histoplasma*, while the tongue ulceration reflects disseminated disease that was previously asymptomatic until immunosuppression following treatment of psoriasis.

Infliximab is associated with the greatest rates of infections with histoplasmosis compared to the other TNF-⍺ inhibitors [8]. Treatment of histoplasmosis associated with TNF-⍺ inhibitors includes cessation of the offending agent and initiation of intravenous amphotericin B followed by oral itraconazole [8,9]. It is uncertain whether long-term antifungal therapy with oral itraconazole should be continued for greater than 12 months [8]. Further uncertainty exists regarding the safety of resuming therapy with TNF-⍺ inhibitors after antifungal therapy [8]. Some note that treatment with TNF-⍺ inhibitors may be safely resumed in patients with undetectable antigen levels and following antifungal therapy [8]. Alternatively, patients may be restarted on alternative immunosuppressive therapy including non-TNF-⍺ inhibitor biologics, disease-modifying anti-rheumatic drugs, and steroids [10]. The clinical stability of our patient’s psoriasis did not signal a need to resume TNF-⍺ inhibitor therapy. Some patients demonstrate clinical worsening when stopping TNF-⍺ inhibitor therapy due to immune reconstitution inflammatory syndrome, which necessitates treatment with prednisone [8].

Currently, no active screening for histoplasmosis is recommended for asymptomatic patients or those without epidemiological risk factors when initiating treatment with TNF-⍺ inhibitors [5]. However, symptomatic patients or those with a recent history of high-risk activities in endemic areas should be screened for active disease with a chest radiograph prior to initiating therapy [5].

The differential diagnosis for this patient presenting with a tongue ulcer and granulomas includes Crohn's disease, sarcoidosis, granulomatosis with polyangiitis, TNF-α inhibitor-induced granulomatous glossitis, and pyoderma gangrenosum.

Crohn's disease is an inflammatory bowel disease characterized by non-necrotizing granulomas and often presents with aphthous ulcers and weight loss [11]. This patient did not report diarrhea or exhibit findings of Crohn's disease on colonoscopy. Additionally, aphthous ulcers demonstrate a neutrophilic infiltrate rather than granulomatous inflammation [11].

Sarcoidosis is characterized by widespread non-necrotizing granulomas associated with lymphadenopathy, lupus pernio, and erythema nodosum [12]. CXR demonstrates bilateral hilar lymphadenopathy and coarse reticular opacities [12]. Ulcerative sarcoidosis is the rarest variant [13]. Sarcoidosis is a diagnosis of exclusion that requires infectious etiologies to be ruled out [12].

Granulomatosis with polyangiitis is a small and medium vessel necrotizing granulomatous vasculitis involving the nasopharynx, lungs, and kidneys with serology positive for cytoplasmic ANCA and proteinase 3-ANCA [14]. CXR may demonstrate large nodular densities. Classic presentation includes hemoptysis, cough, and hematuria [14]. This patient lacked these symptoms, and indirect immunofluorescence was negative for cytoplasmic ANCA and proteinase 3-ANCA.

Patients taking TNF-α inhibitors can develop non-necrotizing granulomatous glossitis [15]. However, this is a diagnosis of exclusion and not compatible with the patient’s pulmonary findings or positive tissue culture for *H. capsulatum*.

Pyoderma gangrenosum may present as an extremely painful ulcer. However, it is a diagnosis of exclusion with no definitive clinical or histopathologic findings [16]. Oral pyoderma gangrenosum is exceedingly rare, so this diagnosis excludes a lesion with a positive fungal culture and supportive findings for histoplasmosis [16].

## Conclusions

Dermatologists considering the use of TNF-⍺ inhibitors for the treatment of psoriasis should exercise vigilance regarding the heightened risk of infections, including histoplasmosis. Thorough pretherapeutic evaluation requires knowledge of exposures and endemic areas that may render patients more vulnerable to infection. This consideration extends to areas beyond the United States, such as Latin America in the case of our patient.

In patients who present with pulmonary or extrapulmonary complaints after initiation of treatment with TNF-⍺ inhibitor therapy, a thorough history and clinical evaluation is necessary to evaluate for histoplasmosis. Diagnosis of histoplasmosis is based on clinical presentation, epidemiology, and appropriate laboratory testing. Treatment involves intravenous amphotericin B followed by oral itraconazole.

## References

[1] Saurat JH, Stingl G, Dubertret L (2008). Efficacy and safety results from the randomized controlled comparative study of adalimumab vs. methotrexate vs. placebo in patients with psoriasis (CHAMPION). Br J Dermatol.

[2] Bakleh M, Tleyjeh I, Matteson EL, Osmon DR, Berbari EF (2005). Infectious complications of tumor necrosis factor-alpha antagonists. Int J Dermatol.

[3] Chirch LM, Cataline PR, Dieckhaus KD, Grant-Kels JM (2014). Proactive infectious disease approach to dermatologic patients who are taking tumor necrosis factor-alfa antagonists: Part I. Risks associated with tumor necrosis factor-alfa antagonists. J Am Acad Dermatol.

[4] Teixeira Mde M, Patané JS, Taylor ML (2016). Worldwide phylogenetic distributions and population dynamics of the genus histoplasma. PLoS Negl Trop Dis.

[5] Hage CA, Bowyer S, Tarvin SE, Helper D, Kleiman MB, Wheat LJ (2010). Recognition, diagnosis, and treatment of histoplasmosis complicating tumor necrosis factor blocker therapy. Clin Infect Dis.

[6] Kamili Qu, Menter A (2010). Atypical presentation of histoplasmosis in a patient with psoriasis and psoriatic arthritis on infliximab therapy. J Drugs Dermatol.

[7] Zattar GA, Cardoso F, Nakandakari S, Soares CT (2015). Cutaneous histoplasmosis as a complication after anti-TNF use--case report. An Bras Dermatol.

[8] Vergidis P, Avery RK, Wheat LJ (2015). Histoplasmosis complicating tumor necrosis factor-α blocker therapy: a retrospective analysis of 98 cases. Clin Infect Dis.

[9] Johnson PC, Wheat LJ, Cloud GA (2002). Safety and efficacy of liposomal amphotericin B compared with conventional amphotericin B for induction therapy of histoplasmosis in patients with AIDS. Ann Intern Med.

[10] Brown RA, Barbar-Smiley F, Yildirim-Toruner C, Ardura MI, Ardoin SP, Akoghlanian S (2021). Reintroduction of immunosuppressive medications in pediatric rheumatology patients with histoplasmosis: a case series. Pediatr Rheumatol Online J.

[11] Lauritano D, Boccalari E, Di Stasio D, Della Vella F, Carinci F, Lucchese A, Petruzzi M (2019). Prevalence of oral Lesions and correlation with intestinal symptoms of inflammatory bowel disease: a systematic review. Diagnostics (Basel).

[12] Judson MA (2015). The clinical features of sarcoidosis: a comprehensive review. Clin Rev Allergy Immunol.

[13] Yoo SS, Mimouni D, Nikolskaia OV, Kouba DJ, Sauder DN, Nousari CH (2004). Clinicopathologic features of ulcerative-atrophic sarcoidosis. Int J Dermatol.

[14] Seo P, Stone JH (2004). The antineutrophil cytoplasmic antibody-associated vasculitides. Am J Med.

[15] Christoforidou A, Goudakos J, Bobos M, Lefkaditis E, Vital V, Markou K (2013). Sarcoidosis-like granulomatosis of the hypopharynx as a complication of anti-TNF therapy. Am J Otolaryngol.

[16] Bissonnette C, Kauzman A, Mainville GN (2017). Oral pyoderma gangrenosum: diagnosis, treatment and challenges: a systematic review. Head Neck Pathol.

